# Editorial: Pathological implications of metabolic and cerebrovascular diseases in neurocognitive disorders

**DOI:** 10.3389/fendo.2024.1367575

**Published:** 2024-01-17

**Authors:** Satoshi Saito, Yoshiki Hase, Masashi Tanaka

**Affiliations:** ^1^ Department of Neurology, National Cerebral and Cardiovascular Center, Suita, Japan; ^2^ Neurovascular Research Group, Translational and Clinical Research Institute, Newcastle University, Newcastle upon Tyne, United Kingdom; ^3^ Department of Rehabilitation, Health Science University, Fujikawaguchiko, Japan

**Keywords:** diabetes mellitus, diabetes-associated cognitive dysfunction, neurocognitive disorders, Alzheimer’s disease, small vessel disease, vascular cognitive impairment

Cerebrovascular dysfunction plays an important role in the development of neurocognitive disorders. Epidemiological studies have underscored the efficacy of tightly managing vascular risk factors as a preventive measure against neurocognitive disorders, emphasising the robust correlation between cerebrovascular disease (CVD) and dementia (1). In this Research Topic, significant contributions have been made through valuable research papers, predominantly examining the pathogenesis of dementia from two aspects: type 2 diabetes mellitus (T2DM) and cerebral small vessel disease (SVD).

Diabetes-associated cognitive dysfunction (DACD) is a complication caused by chronic hyperglycaemia and microvascular diseases, leading to cognitive impairments such as decreased learning ability, memory, attention, and information processing speed. Xu et al. suggested that microglial signalling pathways could be therapeutic targets for DACD based on clinical evidence of cerebral inflammation in T2DM. Microglial activation contributes to neuronal injury and cognitive impairment in T2DM models. Furthermore, microglia enhance synaptic plasticity by expressing and releasing brain-derived neurotrophic factor (BDNF). BDNF drives long-term potentiation in the hippocampus, and altered BDNF levels have been associated with increased appetite, obesity, T2DM, depression, Parkinson’s disease, and Alzheimer’s disease (2). Sumbul-Sekerci et al. measured BDNF levels in the blood of healthy controls and patients with prediabetes or T2DM to investigate the role of BDNF in cognitive impairment in prediabetes and T2DM. However, the mediating role of BDNF in the pathology underlying cognitive impairment in diabetes was not determined. This non-significant finding could be attributed to the limitations of the sample size.

Adults with T2DM have an increased risk of developing specific brain disorders, particularly stroke and dementia. Although these disorders are not commonly categorised as classic microvascular complications of diabetes, increasing evidence suggests that microvascular dysfunction is a crucial underlying mechanism. Ikeda et al. investigated the association between the burden of SVD and stroke recurrence in patients with acute cerebral infarction or transient ischaemic attack. This study revealed a significant correlation between stroke recurrence and high SVD burden, as determined by the total SVD scores defined using magnetic resonance imaging. These findings include lacunae, cerebral microbleeds, moderate-to-severe white matter hyperintensities, or moderate-to-severe perivascular spaces in the basal ganglia. This study contributed to the identification of high-risk patients, thereby facilitating the implementation of preventive measures. Shinohara et al. explored the correlation between the burden of SVD and behavioural and psychological symptoms in patients with mild cognitive impairment or mild dementia. In addition to the total SVD scores, the study emphasised the importance of rating cerebral amyloid angiopathy (CAA)-SVD scores based on the severity of lobar cerebral microbleeds, cortical superficial siderosis, perivascular spaces in the centrum semiovale, and white matter hyperintensities. The modified CAA-SVD scores incorporate additional imaging markers, including posterior dominant white matter hyperintensities and cerebral microinfarcts related to CAA. The presence of behavioural and psychological symptoms were associated with the modified CAA-SVD score and not with the CAA-SVD or total SVD scores. In the same cohort, Matsuda et al. focused on longitudinal changes in SVD severity and cognitive function. They observed that a higher number of lobar cerebral microbleeds and prolonged psychomotor speed at baseline were associated with severe cognitive impairment. Both studies highlight the pivotal role of the SVD burden in neurocognitive disorders.

All papers on this topic have highlighted the pathological significance of metabolic diseases and cerebrovascular changes in the development of dementia. Xiao et al. aimed to clarify the association between diabetes and cognitive impairment using a multiple linear regression model. The authors reported that elevated serum creatinine levels were significantly associated with cognitive impairment. Given the shared microvascular structures of the brain and kidneys, the possibility of common pathophysiological processes in renal dysfunction and cognitive impairment warrants further consideration. Cooke et al. proposed a significant association of psychiatric disorders with brain aneurysms and subarachnoid haemorrhages in US Veterans. Negri et al. conducted a comprehensive review to evaluate the involvement of the transient receptor potential channels in brain microvascular function. Despite the emergence of numerous safe and effective treatments for T2DM in the 21st century, the global prevalence of T2DM is steadily increasing. As Yin et al. highlighted, the pressing need for a better understanding and standardised management of diabetic cognitive dysfunction demands immediate attention. This collection of papers on this Research Topic sheds light on the complex interplay between vascular factors, metabolic conditions, and cognitive health. This deeper understanding highlights avenues for potential improvements in diagnostics, preventive strategies, and therapeutic interventions for neurocognitive disorders.

## Author contributions

SS: Conceptualization, Writing – original draft. YH: Conceptualization, Writing – review & editing. MT: Conceptualization, Writing – review & editing.
